# Assessing real-world implementability of a multimodal group-based tele-prehabilitation program in cancer care: a pragmatic feasibility study

**DOI:** 10.3389/fonc.2023.1271812

**Published:** 2023-10-26

**Authors:** Alexia Piché, Daniel Santa Mina, Sylvie Lambert, Isabelle Doré

**Affiliations:** ^1^ Centre de recherche du Centre hospitalier de l’Université de Montréal, Montréal, QC, Canada; ^2^ School of Kinesiology and Physical Activity Sciences, Faculty of Medicine, Université de Montréal, Montréal, QC, Canada; ^3^ Faculty of Kinesiology and Physical Education, University of Toronto, Toronto, ON, Canada; ^4^ Department of Anesthesia and Pain Management, University Health Network, Toronto, ON, Canada; ^5^ St. Mary’s Research Centre, Montréal, QC, Canada; ^6^ Ingram School of Nursing, McGill University, Montréal, QC, Canada; ^7^ Department of Social and Preventive Medicine, School of Public Health, Université de Montréal, Montréal, QC, Canada

**Keywords:** feasibility, acceptability, fidelity, exercise, nutrition, psychosocial support, education, telehealth

## Abstract

**Introduction:**

Multimodal prehabilitation is intended to optimize a patient’s mental and physical health prior to surgery. Most multimodal prehabilitation interventions are delivered on a one-on-one format, which may limit benefits associated with social interactions that can be achieved in a group context, and are delivered in-person, which may limit the accessibility. The purpose of this study was to develop a group-based, multimodal, tele-prehabilitation intervention for individuals diagnosed with cancer (iACTIF) and assess its implementability in a “real-world” clinical setting by measuring feasibility, acceptability, fidelity, and preliminary effects.

**Methods:**

A prospective, single-group, pragmatic feasibility study was conducted with assessments at baseline, pre-surgery, and 12-weeks post-surgery. iACTIF consisted of three 90-min live videoconference sessions per week, including exercise and educational components. Descriptive statistics were used to document feasibility, acceptability, and fidelity indicators. Paired t-test, Wilcoxon test, and Cohen’s D-test were conducted to assess changes in health-related outcomes.

**Results:**

A total of 25 participants (mean age ± SD= 60.2 ± 14.0) were recruited. The feasibility assessment revealed a low referral rate (31.4%) and a high study retention (98%) and program attendance [session attended/possible session] (70.2%), with a prehabilitation window of 32.7 days (SD= 20.9, median= 28). Acceptability was high (84%–100%) according to satisfaction, utility and safety, delivery modality, and intention to continue physical activity and to recommend iACTIF to a relative. Pre–post-intervention assessments suggest positive changes on physical functional capacity based on the 2-min step test (mean difference= +18.9 steps, p=0.005), the 30-s sit-to-stand (mean difference= +1.1 repetition, p=0.011), and volume of moderate intensity physical activity per week (mean difference= +104.8 min, p<0.001). Fidelity was supported by conformity and coherence, with only minimal adjustments required to meet participants’ needs.

**Discussion:**

iACTIF implementability in a “real-world” clinical setting is promising, and preliminary outcomes suggest moderate benefits on physical health and small increase in mental health indicators.

## Introduction

Exercise before, during, and after cancer treatment is a safe and effective strategy that provides numerous physical and mental health benefits for people diagnosed with cancer (1–4). Recent studies show that the earlier exercise is introduced, including immediately after a cancer diagnosis, the greater the benefits are for the patient (5). Prehabilitation uses the window of opportunity between cancer diagnosis and surgery (or initiation of treatments) to optimize physical and psychological functioning. Prehabilitation can mitigate preoperative patient deconditioning and prevent or reduce the incidence and severity of symptoms associated with the cancer diagnosis and treatment (i.e., stress, anxiety, depressive symptoms, pain, fatigue) and possible treatment-related complications, while accelerating recovery after surgery (6–8). Prehabilitation that includes two or more intervention components is referred to as multimodal prehabilitation, and typically comprises exercise, nutrition, psychosocial support, and/or a behavioral intervention (9). The synergistic relationship between these modalities is intended to optimize outcomes and address the various adverse health effects of cancer and its treatment (9, 10). However, intervening within the short window of opportunity between cancer diagnosis and surgery represents a challenge. The main barriers to intervening in the prehabilitation window include accessibility (i.e., limited program availability and transport and parking fees for in-person program), lack of time (i.e., many medical appointments and some patients are still working), lack of motivation, and cancer-related side effects such as pain, fatigue, or anxiety and depressive symptoms (11–14).

Group-based exercise can enhance motivation to initiate and maintain behavior change and increase social interactions, social support, and sense of belonging for people diagnosed with cancer (15–17). Compared to unsupervised interventions, supervised exercise interventions have shown to be more effective and provide greater benefits on physical function, quality of life, anxiety, and depressive symptoms (1, 18). Supervision contributes to increased self-efficacy and a sense of safety and, thus, promotes continued exercise participation when then transferring to an unsupervised setting, such as home (18). A growing body of literature has described the feasibility and potential benefits of multimodal tele-prehabilitation interventions delivered in a one-on-one supervised setting for people with cancer (19–24).

Telehealth multimodal prehabilitation interventions, including those with an exercise component, have been shown to be feasible and acceptable among patients with cancer (19–21) and health care professionals (23, 24). Tele-rehabilitation has also shown promise in increasing physical activity (25) and reducing specific symptoms (26) among patients with cancer. Telehealth interventions can help reduce inequities regarding intervention accessibility for people living in remote areas (21, 23, 24, 27); offer an alternative for individuals who have difficulty traveling due to their health condition, limited resources, and work-related or other obligations (21); and could improve compliance with appointments, reduce the number of in-person clinic visits, and potentially, reduce the burden on caregivers (28). It is unknown whether a multimodal prehabilitation program could be delivered in a telehealth group-based format that might offer the advantages of social support described above. To address these limitations, we developed iACTIF, the first telehealth, group-based, multimodal prehabilitation program for individuals diagnosed with cancer. While no previous study has delivered or evaluated prehabilitation in this format, the broader literature supports effectiveness of in-clinic prehabilitation (6–8), acceptability, and feasibility of tele-prehabilitation interventions (20, 21, 24, 29–31), and strongly recommends supervised (1, 18, 19, 32) and group-based (15–17) approaches for people diagnosed with cancer. It appears reasonable to extrapolate from these findings to examine the effect of a prehabilitation intervention that addresses main barriers to participation.

To examine iACTIF, we employed a pragmatic strategy (33) and an implementability framework (34) to facilitate the translation of the research intervention into a “real-world” clinical practice. Implementability refers to “the likelihood that an intervention will be adopted into routine practice and into health consumer behaviors across settings and over time” (34). Accordingly, we aimed to offer findings that relate to a “real-world” usual care setting to inform whether iACTIF could be adopted into clinical contexts (35). The specific objectives of this study were to assess the following: i) feasibility, the extent to which the intervention can be carried out successfully in a “real-world” usual care setting (34, 36); ii) acceptability, the extent to which the intervention is considered appropriate, satisfactory, or attractive by program recipients (34, 36, 37) including benefits on patient health-related outcomes; and iii) fidelity, the extent to which the intervention was implemented as planned and adaptations were made (38).

## Methods

### Study design

We conducted a prospective, single-group, pragmatic feasibility study. According to the Conceptual Framework of Implementability of Healthcare Interventions (34) guiding this study, to determine whether an intervention has the potential for scaling-up and for sustainability, acceptability, fidelity, and feasibility—including effectiveness—of the intervention needs to be considered early on, during the preliminary phases of intervention development, evaluation, and implementation. This approach facilitates the adoption of an intervention in the standard of care in “real-world” clinical setting. The study was approved by the Research Ethics Board at CHUM (no. 21.021). Participation was voluntary, and all participants provided written informed consent prior to participation.

### Setting

iACTIF was offered to patients recently diagnosed with cancer at the Centre Hospitalier de l’Université de Montréal (CHUM) Integrated Cancerology Center (CICC) in Montreal, Canada, in close partnership with the Virage Foundation who provides kinesiology services for CHUM patients since 2013 and supervised all the iACTIF group sessions within their usual clinical offer.

### Participants

Patients were eligible for iACTIF if they i) were 18 years and over, ii) had a cancer diagnosis, iii) were receiving a cancer surgery in 2 weeks or more, iv) had medical authorization for exercise, v) were able to read and understand French, vi) had access to an Internet connection *via* a device with a camera (cellphone, computer, tablet, etc.), and vii) had the knowledge (or help) required to connect to the live videoconference group sessions. Patients receiving neoadjuvant treatments were excluded and referred to the during-treatment kinesiology program at the Virage Foundation.

### Recruitment procedures

Patients could be self-referred or referred by their healthcare professional *via* the electronic hospital referral system. Study promotion and recruitment strategies included targeted emails sent to the head manager of the CICC and healthcare professionals, and presentation of the program at a nurses’ meeting, posters, and digital monitor advertisements in waiting rooms of the CICC. Promotional material included a brief description of the program, targeted population, and indicated referral procedures to the Virage Foundation kinesiology service. If patients declined to enroll in the study, they could still access the tele-prehabilitation program with the Virage Foundation.

### Intervention

iACTIF comprised three 90-min sessions per week delivered using synchronous videoconferencing technology with Zoom software (Zoom Video Communications, San Jose, CA). The program was free, and no equipment was provided or needed; participants could purchase resistance bands through the Virage Foundation, if desired. Since we used a rolling recruitment strategy, the number of participants and the group composition changed continuously as new participants entered and others left for surgery. The duration of the intervention varied for each participant depending on their waiting time for surgery. A minimum of two participants was needed to deliver a session, and no more than 10 participants per session were included to ensure adequate monitoring for safety. Three certified kinesiologists specialized in exercise and cancer from the Virage Foundation were involved in the group supervision (two main kinesiologists and one substitute): two of them held a graduate degree, had 8 years of experience with cancer population and working as a team; the other one was pursuing a graduate degree, had 1 year of experience with cancer population and working in the team. Each group session was supervised by two kinesiologists: one leading the session and the other monitoring for safety. Each participant provided their home address and phone number and were advised to notify the kinesiologist if they were alone at home at the start of the group session in case of adverse event.

Each session included exercise and education components. The 60-min exercise component included the following: i) a 10-min warm-up (including mobility exercises and progressively increasing the intensity with dynamic exercises); ii) a circuit of nine exercise stations targeting muscle strengthening, aerobic endurance, and balance (see **Table 1**); and iii) a 10-min cool down (including flexibility exercises). Each station targeted a specific muscle group, cardio or balance, but exercise prescription was individualized for every participant during the initial telehealth fitness assessment (further details provided below). Every exercise station lasted 2 min, with an effort time from 30 s to 90 s depending on the participant’s condition at the initial telehealth fitness assessment (with 30–90 s of rest between exercise stations). Effort time could be revised by the kinesiologist to foster progress throughout the sessions. Effort intensity was monitored with the 10-point Borg Rating of Perceived Exertion (RPE) scale (39), targeting 3 to 5 (moderate) to ensure the safety of participants at home. Participants were asked to adjust their camera, so the kinesiologists could see their entire body at all times. Following the exercise component, participants engaged in a 30-min educational session including a 10-min teaching vignette followed by a 20-min group discussion guided by the health professional—a kinesiologist, nutritionist, or psychologist, all specialized in oncology—assigned to the session educational theme. A total of nine educational vignettes have been developed under four main themes: exercise, nutrition, psychological support, and sleep.

**Table 1:** Exercise stations of the circuit.

#	Exercise Type/Muscle Group	Examples
**1**	Cardio	High knees, jumping jacks, fast feet
**2.**	Lower body	Chair squat, standing squat
**3.**	Upper body—push	Wall push ups, resistance band push
**4.**	Cardio	High knees, jumping jacks, fast feet
**5.**	Balance	Tandem or single leg stand
**6.**	Cardio	High knees, jumping jacks, fast feet
**7.**	Upper body—pull	Horizontal arm abduction or rowing
**8.**	Abdominals	Dead bug, wall plank, plank
**9.**	Glutes	Bridge, standing hip extension

Targeted intensity was moderate (3–5 on the Borg CR10 Scale) (Borg 1982). Progression was made by increasing the difficulty level of the exercises or the station effort duration.

### Data collection

Data were collected at three time points: T1 (baseline), T2 (post-intervention), and T3 (follow-up).

#### Implementability

Feasibility was measured from T1 to T2 by i) total number of referrals over the recruitment period and referral rate [average number of referrals/week], ii) eligibility rate [eligible participants/total referrals], iii) recruitment rate [recruited participants/eligible referrals], iv) study retention, v) prehabilitation window [time since referral to surgery] and duration [time since T1 to surgery], vi) attendance [number of sessions attended/number of possible sessions—based on the participant specific prehabilitation duration], and vii) obstacles encountered and strategies used to overcome these obstacles.

Acceptability measures were introduced at T2 and included i) satisfaction of the intervention content and modalities, ii) perceived utility, iii) perceived safety, iv) future intentions to practice physical activity, v) intention to recommend the prehabilitation intervention to a loved one, and vi) sense of belonging to the group. Questions for all acceptability indicators, except sense of belonging, were developed by the team based on the Patient Satisfaction Questionnaire Short-Form (PSQ-18) (40, 41). Participant satisfaction was described across five categories: general (four items), exercise component (three items), educational component (three items), telehealth format (three items), supervision (two items), and perceived usefulness (two items). The perceived safety, future intentions to maintain physical activity, and intentions to recommend to a loved one were measured with one item. All questions were measured using a 5-point Likert scale (totally agree, agree, neutral, disagree, and totally disagree) (40); agree and totally agree were dichotomized to represent an acceptable level of satisfaction, perceived utility, safety, and intentions for physical activity. The number of falls was also collected for safety outcomes. The Relatedness to Others in Physical Activity Scale (ROPAS) is a valid and reliable tool and was used to assess sense of belonging (42). A written open-answer question was added at the end of the satisfaction questionnaire to collect overall comments and suggestions on the program.

Fidelity was measured from T1 to T2 in a logbook completed by the research assistant (RA) to assess i) session conformity (how well were all the intervention parameters delivered as planned) by documenting the structure of the group sessions (time allowed, warm up, circuit, and cool down) and educational content (time allowed, delivery of the content, discussions), and ii) session consistency (type and volume of adaptations required to ensure the appropriate intervention delivery) over time and between kinesiologists by assessing structure of the sessions, and exercise and educational content (34, 38). At T3, supplementary fidelity data were collected in a one-hour semi-structured single group interview with the three kinesiologists who delivered iACTIF. The interview was conducted by the first author (AP) and the study principal investigator (ID) with the three kinesiologists. A semi-structured interview guide was developed to gather information to complement logbook information (provider qualifications, work experience, conformity of the intervention, and consistency in delivery over time and between kinesiologists). This interview was recorded and transcribed verbatim.

#### Health-related outcomes

T1 assessments took place as soon as possible following receipt of the referral (minimum 2 weeks before surgery); a delay of one to three working days was planned to confirm the eligibility of the participant, obtain consent, and proceed to the T1 telehealth fitness assessment by a certified kinesiologist. All participants performed functional tests, practiced the positioning of the camera, familiarized with the prescribed exercises, and received explanations for the group sessions. Physical function was measured *via* the 2-min step test (43)—the number of steps at a targeted height (mid-point between patella and iliac crest) in 2 min—and the 30-s sit-to-stand test (44)—the number of full stands in 30 s starting in seated position with arm crossed on the chest; where an increase in repetitions for both tests is favorable. Both tests have demonstrated reasonable reliability and validity in a telehealth setting (45). Weekly physical activity volume was measured during the telehealth fitness assessment; the kinesiologist asked the participants about their recent practice of physical activity focusing on the frequency, intensity, time (duration), and type of physical activity (FITT). Self-reported questionnaires were provided in French and completed on REDCap (12.2.1, ^©^ 2022 Vanderbilt University) to assess stress level, anxiety and depressive symptoms, health-related quality of life, social support, and sociodemographic and clinical profile. Participants’ stress level was measured using a single item assessing the amount of stress in one’s life rated on a 5-point Likert scale ranging from not at all stressful to extremely stressful (46). The measure is commonly used in large surveys, including Statistics Canada (46). Anxiety and depressive symptoms were measured using the French version of the Hospital Anxiety and Depression Scale (HADS) (47). The psychometric properties of HADS have been assessed in various populations and the French–Canadian version shows good reliability and validity (48). Health-related quality of life was measured using the EORTC-QLQ-C30 questionnaire (49). The psychometric properties were evaluated first with lung cancer patients (49) and then with many types of cancer (50); the EORTC-QLQ-C30 shows good validity and reliability (51). Participants’ perceived social support was measured using the Medical Outcome Study-Social Support Survey (MOS-SSS) (52–54), a multidimensional measure of social support validated for people with cancer (55). Sociodemographic (gender, age, ethnicity, aboriginal status, racial group, postal code, household composition, education, employment status, and household income) and clinical (type of cancer, date of diagnosis and surgery, type planned treatment, risk factors for cardiovascular disease, other diseases, and BMI) data were also collected through the online self-reported questionnaire.

The T2 assessment took place 1–3 days prior to surgery. Functional tests conducted at T1 in the telehealth fitness assessment were repeated at T2, and self-report measures except the sociodemographic and clinical questions, the MOS-SSS, and the EORTC-QLQ-C30 to reduce participation burden.

The T3 assessment took place 12 weeks after surgery. Functional tests of the telehealth fitness assessment were the same as T1 and T2. T3 online self-reported questionnaire was the same as T1, without sociodemographic questions and adding a question on rehabilitation participation asking participants: currently, are you participating or are you registered in a rehabilitation program (post-treatment physical activity program at the CHUM or elsewhere)?

### Data analysis

Descriptive statistics (frequency, percentage, mean, median, standard deviation, and range) were used for acceptability and feasibility indicators. Paired T-test and Wilcoxon signed-rank test were performed to explore changes between T1 and T2, and T1 and T3. Cohen’s D effect size was calculated and interpreted as small (d = 0.2), medium (d = 0.5), and large (d = 0.8) based on benchmarks suggested by Cohen (56). Statistical analyses were performed in R software version 4.0.4 and R Studio version 4.0.2. Qualitative data were composed of the verbatim transcript of the group interview with the three kinesiologists (fidelity) and the written answers to the open-answer question in the satisfaction questionnaire (acceptability). Simple deductive content analysis was performed to categorize the group interview’ verbatim and the participants’ comments and suggestions regarding the program. In an Excel sheet, the first author reduced and condensed the data to eliminate the superfluous elements before attributing codes to the different meaning units, identifying patterns of similar codes, and categorizing those patterns under categories of relevant meaning. Qualitative findings were integrated into the results section to complement quantitative results where appropriate.

## Results

### Feasibility

**Figure 1:**
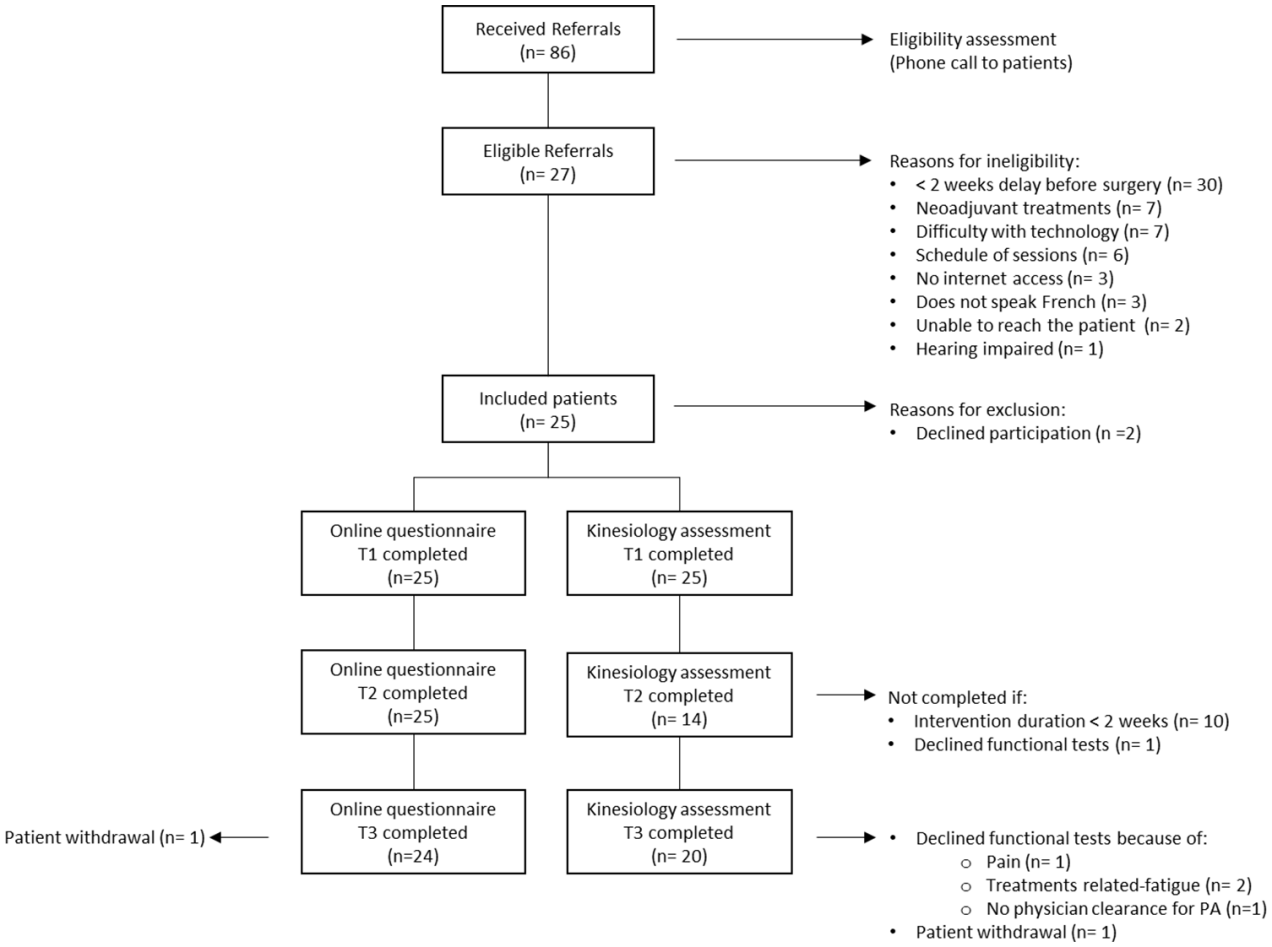
Study flow diagram.

**Figure 1** provides the participant flow diagram. A total of 86 referrals were received during the 7-month recruitment period from June to December 2021. The weekly referral rate (mean (SD)) was 3.0 (2.5) over the recruitment period. All referrals received were from healthcare professionals. Among the referred patients, 27 (31.4%) were eligible, and among them, 25 (92.6%) were enrolled in iACTIF and consented to participate in the study. The main reasons why participants were not eligible were related to the timing of the referral (surgery in < 2 weeks) (n=30), ongoing neoadjuvant treatments (n=7), technology issues (n=7), and availability at the proposed schedule of the intervention (n=6).

All participants completed the online questionnaires at T1 and T2, and only one refused to complete T3 without giving any reason. All participants completed the telehealth fitness assessment at T1, 14 participants at T2, and 20 participants at T3. The prehabilitation window was shortened for some participants (n=10) who underwent surgery faster than expected, preventing data collection at T2. Participants were, on average, 60.2 years (SD = 14.0, range= 24 to 78) and predominantly female (80%), and the most common diagnosis was breast cancer (68%). Detailed sociodemographic and clinical characteristics are presented in **Table 2**. Participants attended an average of 6.5 (SD= 4.6, median = 6,0) sessions over a prehabilitation window of 32.7 (SD= 20.9, median=28.0) days. The mean attendance was 70.2% (SD=24.3). Detailed feasibility outcomes are shown in **Table 3**.

**Table 2:** Baseline (T1) characteristics of participants (n = 25).

Characteristics	*n* (%) or mean (SD)
Age (years), mean (SD)	60.2 (± 14.0)
Sex, female n (%)	20 (80)
First nation status, yes n (%)	0 (0)
Ethnicity, white/Caucasian n (%)	23 (92)
Matrimonial status, n (%)	
Single	11 (44)
Married/common-law	10 (40)
Divorced/separated	2 (8)
Widow	2 (8)
Household composition, n (%)	
Alone	13 (52)
With spouse or husband without children	8 (32)
With spouse or husband and one or more children	1 (4)
With one or more other family member(s) (i.e.: brother, sister, parent)	2 (8)
With a friend(s) or roommate(s)	1 (4)
Education, n (%)	
No degree	1 (4)
High school	3 (12)
Professional	2 (8)
College	4 (16)
University—1st cycle	11 (44)
University—2nd and 3rd cycles	4 (16)
Employment status, n (%)	
Part time or full time	4 (16)
Unemployed (regardless of reason)	1 (4)
Retired	15 (60)
On temporary leave (e.g., sickness, maternity/paternity, work accident)	5 (20)
Household income, n (%)	
<50,000$	8 (32)
50,000–99,999$	8 (32)
>99,999$ $	5 (20)
I do not know	4 (16)
First cancer diagnosis, yes n (%)	21 (84)
Cancer type n (%)	
Breast	17 (68)
Prostate	2 (8)
Liver	2 (8)
Colorectal	1 (4)
Bladder	1 (4)
Mouth	1 (4)
Vulva	1 (4)
Cardiovascular disease risk factors, n (%)	
0	7 (28)
1	4 (16)
2	5 (20)
3+	9 (36)
Other health problems, n (%)	
0	2 (8)
1	7 (28)
2	6 (24)
3+	10 (40)
BMI, mean (SD)	27.9 (± 8.5)
BMI categories, n (%)	
Insufficient weight, <18.5	0 (0)
Normal weight 18.5–24.9	12 (48)
Weight excess, 25.0–29,9	7 (28)
Obesity, class I, 30.0–34,9	5 (20)
Obesity, class II, 35.0–39,9	0 (0)
Obesity, class III, ≥40.0	1 (4)

**Table 3:** Feasibility outcomes.

Measures	n (%) or mean ± SD	Median
Weekly referral rate	3.0 **±** 2.5	2
Monthly referral rate	12.3 **±** 3.3	13
Eligibility rate (eligible referrals/total referral)	27 (31.4)	–
Total recruitment rate (recruited/total referral)	25 (29.1)	–
Eligible recruitment rate (recruited/eligible referrals)	25 (92.6)	–
Study retention	24 (96)	–
Attendance rate (session attended/possible session)	70.2 **±** 24.3	70
Prehabilitation window in days (time referral–surgery)	32.7 **±** 20.9	28
Prehabilitation duration in days (time T1–surgery)	27.0 **±** 19.0	24

- Not applicable.

### Acceptability

The responses to the satisfaction survey indicated that all participants were satisfied with the program, were comfortable with the exercise structure, felt safe, and agreed that the supervision of the kinesiologist gave them confidence. No fall occurred during exercise sessions. Sense of belonging (ROPAS) to the group was 4.1 ±1.3. Only few participants would have preferred to alternate telehealth and in-person sessions (16%), to do all the group sessions in person with a kinesiologist (8%), or to have individual sessions supervised with a kinesiologist (12%). The afternoon schedule of the group session did not suit 40% of the participants. The open-ended question suggests that for younger participants who were still working full time before surgery and some older retired participants, this schedule interrupted their afternoon, and they would have preferred morning sessions. Detailed acceptability indicators are presented in **Table 4**.

**Table 4:** Acceptability outcomes for each indicator items.

Indicators	Totally agree + agree n (%)
Satisfaction	
Overall program	
* I am satisfied with the prehabilitation program*	25 (100)
* I had fun*	25 (100)
* The duration of the group sessions suited me*	25 (100)
* The schedule of the group sessions suited me*	15 (60)
Exercise	
* I was comfortable with the exercise circuit structure*	25 (100)
* The level of difficulty of the exercises was adapted to my physical condition*	25 (100)
* The progression in the level of difficulty suited me*	23 (92)
Education	
* The duration of the capsules was adequate*	22 (88)
* The time allowed for the group discussions was adequate*	22 (88)
* The lessons were easy to understand*	23 (92)
Virtual	
* I would have liked to alternate virtual AND in-person sessions with kinesiologists*	4 (16)
* I would have preferred to do all the sessions in person with the kinesiologists*	2 (8)
* I found it easy to connect to the Zoom platform*	21 (84)
Supervision	
* The presence and supervision of kinesiologists gave me confidence*	25 (100)
* I would have preferred one-on-one supervised sessions with a kinesiologist*	3 (12)
Perceived Utility	
*The prehabilitation program helped me prepare well for the surgery*	22 (88)
*I learned things that are useful to me*	23 (92)
Safety	
*At all times, I felt safe*	25 (100)
Number of fall = 0	25 (100)
Intentions for Physical Activity	
*I plan to continue doing physical activity after my surgery*	25 (100)
Intentions to Recommend	
*I would recommend this program without hesitation to a loved one*	25 (100)
Sense of Belonging (ROPAS), range 1–6	mean (SD)
*I feel like I have developed a close bond with others*	3.2 (1.6)
*I feel like I fit in well with others*	4.8 (1.3)
*I feel like I am included by others*	4.5 (1.3)
*I feel like I am part of a group who share my goals*	4.5 (1.4)
*I feel like I am supported by others in the prehabilitation group*	4.0 (1.5)
*I feel like others want me to be involved with them*	3.7 (1.3)
Total score	4.1 (1.3)

At T3, the majority of participants were already participating (58%) or registered (16%) in a rehabilitation program with the Virage Foundation. Reasons for not being involved in a rehabilitation program 3 months after surgery included not being interested (8%), could not participate because of work schedule (4%), not eligible because they were living in a remote area not covered by the Virage Foundation (8%), or not yet having the authorization from their oncologist to participate in physical activity (4%).

### Fidelity

According to the interview with the kinesiologists, intervention conformity was observed because the intervention was delivered as planned, and consistency was observed as the intervention structure (i.e., warm-up, circuit, cool down, and educational content) did not vary between providers. The intervention was slightly adapted (see **Table 5**) over time to better meet patients’ needs and preferences. When a change was proposed, all kinesiologists discussed it to ensure full agreement and understanding. Changes were made at the same moment for all kinesiologists. Providers’ training supports the quality and fidelity of the intervention.

**Table 5:** Adaptations to the program.

Component	Adaptations
**Overall program**	▪ Add a one-on-one virtual orientation session after the initial assessment (T1) to review and familiarize with the program’ structure and exercises, especially among those who had no experience in physical activity to facilitate group integration. ▪ Send email with a PDF version of the prescribed exercises when requested by the participant to help them follow the group session.
**Virtual**	▪ Call participants having difficulties to connect to the Zoom^©^ platform.
**Supervision**	▪ To optimize our resources, it was decided that only one kinesiologist was needed to deliver the group session after 1 month of familiarization with the program. Consequently, the first kinesiologist supervised the Monday–Wednesday sessions, the second one the Friday sessions, and the third one was a substitute when needed.
**Education**	▪ The education component was moved at the end of the sessions after 8 weeks of intervention for administrative reasons. ▪ The group discussions never lasted 20 min (more 5–10 min maximum), so the total sessions length was more 75 min rather than the original 90 min planned. ▪ The educational component was reorganized to maximise content delivered to participants. Initially, there was 1 vignette per week, but we changed it to two vignettes per week (Monday–Wednesday) and a vignette recap on Friday.
**Exercise**	▪ A 10th exercise station specific to surgery was added in the first week of delivery to better individualize the exercises.

### Health-related outcomes

Change in health-related outcomes over time are presented in **Table 6**. Based on Cohen’s benchmarks, a slight decrease was observed from T1 to T2, in measures of stress (meanDif [95%CI]: −1.00 [−1.00–3.99]), anxiety (meanDif [95%CI]: −0.88 [−2.00–0.24]), and depressive symptoms (meanDif [95%CI]: −0.50 [−2.50–1.00]). A large increase was observed in the 2-min step test (meanDif [95%CI]: 23.54 [8.42–38.66]), a moderate increase in 30-s sit-to-stand (meanDif [95%CI]: 1.62 [0.44–2.79]), and a large increase in the moderate intensity physical activity volume per week (meanDif [95%CI]: 150.00 [120.00–180.00]). From T1 to T3, a slight decrease was observed in the stress level (meanDif [95%CI]: −1.00 [−1.50–−0.50]), a moderate decrease in the global health status (meanDif [95%CI]: −8.68 [−17.20–−0.16]), and a slight increase was observed in the moderate (meanDif [95%CI]: 120.00 [−90.00–150.00]) and low (meanDif [95%CI]: 51.33 [−33.72–136.38]) intensity physical activity volume per week. Due to the limited sample size, the current study was not adequately powered to identify statistically significant associations. Therefore, p-values are presented in **Table 6** only for indicative purpose and should be interpreted with caution.

**Table 6:** Patient health-related outcomes measures at each time points, mean differences, and effect sizes.

Outcome Measures	Time Point	n	Mean (SD)	Difference in Means with T1 (95% CI)	*p*-value	Cohen’s d
2-min step test	T1	22	98.91 (26.81)	–	–	–
	T2	13	117.77 (14.35)	23.54 (8.42, 38.66)	0.005	0.817
	T3	18	109.5 (22.98)	7.23 (7.41, 21.96)	0.310	0.421
30-s sit-to-stand	T1	22	9.82 (2.44)	–	–	–
	T2	13	10.92 (1.80)	1.62 (0.44, 2.79)	0.011	0.495
	T3	19	9.84 (2.34)	0.06 (−0.95, 1.06)	0.908	0.010
Stress level single item	T1	25	3.16 (0.99)	–	–	–
	T2	25	2.88 (1.01)	−1.00 (−1.00, 3.99)	0.095†	0.280
	T3	24	2.63 (0.97)	−1.00 (−1.50, −0.50)	0.008	0.547
HADS – Anxiety symptoms	T1	25	7.80 (3.12)	–	–	–
	T2	25	6.92 (3.49)	−0.88 (−2.00, 0.24)	0.118	0.266
	T3	24	7.04 (3.98)	−0.71 (−1.84, 0.42)	0.208	0.212
HADS – Depression symptoms	T1	25	4.84 (3.77)	–	–	–
	T2	25	4.28 (3.60)	−0.50 (−2.50, 1.00)	0.490†	0.152
	T3	24	5.17 (3.70)	0.21 (−1.36, 1.78)	0.786	0.087
HADS – Total score	T1	25	12.64 (6.20)	–	–	–
	T2	25	11.20 (6.46)	−1.44 (−3.22, 0.34)	0.109	0.227
	T3	24	12.21 (7.12)	−0.50 (−2.87, 1.87)	0.667	0.065
EORTC-QLQ-C30 –Functional scales	T1	25	74.12 (18.82)	–	–	–
	T3	24	69.33 (17.63)	−6.10 (−13.90, 1.71)	0.120	0.262
EORTC-QLQ-C30 –Global health status/ QoL	T1	25	66.67 (16.67)	–	–	–
	T3	24	57.99 (22.32)	−8.68 (−17.20, −0.16)	0.046	0.442
EORTC-QLQ-C30 – Symptom scales	T1	25	16.81 (13.70)	–	–	–
	T3	24	20.34 (11.39)	4.84 (−0.52, 10.19)	0.075	0.280
MOS-SSS - Social support	T1	25	68.26 (21.16)	–	–	–
	T3	24	68.71 (20.04)	−0.61 (−6.12, 4.94)	0.823	0.006
VPA Volume	T1	15	16.33 (63.26)	–	–	–
	T2	15	0.00 (0.00)	(NA)	1.00†	0.365
	T3	15	0.00 (0.00)	(NA)	1.00†	0.365
MPA Volume	T1	15	28.00 (108.44)	–	–	–
	T2	15	160.00 (58.55)	150.00 (120.00, 180.00)	0.001†	1.515
	T3	15	50.00 (65.79)	120.00 (−90.00, 150.00)	0.396†	0.245
LPA Volume	T1	15	123.00 (161.29)	–	–	–
	T2	15	120.00 (156.02)	−44.10 (−162.50, 240.00)	0.554†	0.019
	T3	15	174.33 (157.30)	51.33 (−33.72, 136.38)	0.216	0.322

HADS, Hospital Anxiety and Depression Scale; EORTC-QLQ-C30, European Organisation for Research and Treatment of Cancer—Quality of Life Questionnaire—Core Questionnaire; MOS-SSS, Medical Outcomes Study Social Support Survey Instrument; VPA, Vigorous Physical Activity; MPA, Moderate Physical Activity, LPA, Low Physical Activity. p-values are presented only for indicative purpose and should be interpreted with caution given the limited statistical power due to sample size. ^†^Wilcoxon signed-rank test.- Not applicable.

## Discussion

This study is introducing a new prehabilitation format. Considering that individualized exercise interventions are resource intensive (57), group-based interventions are more resource conscientious and can provide greater reach of patients at the same time (57). The pragmatic approach of this study allows to consider the “real-world” clinical setting and accelerates the transfer of scientific knowledge to clinical practice. Using the Conceptual Framework of Implementability of Healthcare Interventions (34) to assess ACTIF, we found high acceptability, feasibility, and fidelity, which support future scalability of the intervention. The pragmatic approach allowed to consider all constraints of the typical clinical setting. The high acceptability of the intervention observed in the present study is consistent with the literature for similar interventions (20, 30, 58–60). Supervision of the session by a kinesiologist appeared to be a real asset in building participant’s confidence for exercise. Other studies mentioned that participants valued the weekly follow-ups with the kinesiologists (11, 22). Moreover, all participants felt safe at all times and no falls occurred, which is comparable to other tele-prehabilitation studies (20, 31). Despite the fact that participants in the group changed regularly, the presence of a great sense of belonging to the tele-prehabilitation group was observed, similar to what has been observed in other prehabilitation and rehabilitation interventions (11, 61). The Zoom platform appeared to have been an appropriate tool, as the majority of the participants were satisfied with the telehealth format and found the platform easy to use. These results align with the literature suggesting that tele-prehabilitation or tele-rehabilitation interventions are well accepted by patients (20, 30, 60). A few studies argue that a small proportion of patients would prefer face-to-face consultations over telehealth consultations (30, 60), which is also consistent with our results indicating that two participants would have preferred to have only face-to-face sessions with kinesiologists.

According to a systematic review on barriers and facilitators to physical activity in individuals diagnosed with cancer, not having enough knowledge about physical activity is a significant barrier to adopting and maintaining physical activity (14). Nearly all participants of our study reported that the educational vignettes were useful and easy to understand and helped prepare for surgery. All participants in our study intended to continue regular physical activity after surgery, which is consistent with Crevenna and colleagues (2021), supporting that participating in prehabilitation would be conducive to participation in post-surgical rehabilitation (62).

While there is no consensus regarding the ideal duration of a prehabilitation intervention, several studies suggest a duration of 4 weeks to achieve minimal benefits (6, 63, 64), but others argue that 3 weeks (65), and even 2 weeks, could be sufficient (66, 67) to observe health benefits. Participants in iACTIF had a mean intervention duration of 27.0 ±19.0 and found it acceptable. The excellent retention rate in iACTIF is consistent with previous studies (68, 69). Attendance to physical activity sessions in a prehabilitation intervention is often a challenge given the short window of opportunity (11). Here, attendance to group sessions (70%) is slightly below but similar to what was observed by Piraux et al. (2020) (77%) who tested the effect of telehealth aerobic, resistance, and inspiratory muscle training over 2–4 weeks in 22 people with esophagogastric cancer.

The main barrier to feasibility was the low eligible referral rate (31%), mostly attributable to the fact that patients were referred <2 weeks prior to their surgery. This concern aligns with literature suggesting that referral procedures to physical activity programs is not part of the standard of care for many physicians (70) and that a common pathway for referral to exercise programs needs to be developed in many clinical settings (70, 71). Addressing this barrier will be paramount before scalability.

The preliminary effects suggest a moderate to large improvement in physical functional capacity and physical activity weekly volume between T1 and T2. Similar results are observed in the telehealth prehabilitation literature for physical functional capacity (20, 22, 31, 58) and weekly moderate physical activity intensity volume (58), and in the in-person prehabilitation literature for physical functional capacity (72). The small improvement in mental health from T1 to T2 is consistent with previous studies (20, 63, 73, 74). From T1 to T3, the moderate deterioration in global health status and small deterioration of the functional and symptoms scales could be attributable to the inevitable physical deconditioning induced by surgery (73) and the chemotherapy or the radiation therapy treatment that some participants had started following surgery (9). However, the literature does suggests that functional deconditioning is generally less severe following these treatment among patients engaging in prehabilitation compared to does who not or even to those who participate in post-operative rehabilitation alone (9, 63, 73, 75). Social support remained unchanged between T1 and T3, and this differs from other research (76). However, no component of the intervention specifically targeted social support other than the group-based modality.

### Limitations and strengths

The variability of the prehabilitation window meant that participants did not have access to the same educational content, which could have influenced their satisfaction and effects outcomes. The prehabilitation window was shortened for some participants (n=10) who underwent surgery faster than expected, limiting data collection at T2. No measure of quality of life or social support was included in the post-intervention assessment (T2) to reduce participant burden, which limits our ability to assess change immediately following the intervention. The sample was predominantly white (92%), which does not allow for the generalization of observations to other cultures or ethnicities. Information about participants’ physical activity level before the cancer diagnosis was not collected. Considering that most participants in our sample had an interest or openness to physical activity, which is not representative of all patients diagnosed with cancer, this could have influenced the outcomes of the present study and should be considered in future study. Adherence to the circuit exercise prescription was not measured in the current study but should be considered in future study. Participants had access to a reliable Internet connection, which represents an accessibility limitation based on socioeconomic status and limits generalizability. This study is innovative by introducing a new prehabilitation format, combining multimodal components, group, and telehealth settings. Given the absence of a control arm, we cannot directly infer cause and effect relationships between our intervention and the outcomes; thus, future studies should employ a randomized controlled trial design to clarify the relationship between the intervention and the observed effects on patient-related outcomes and functional capacity, although the trend of the findings in the current paper appears consistent with the general prehabilitation literature. Future studies should also investigate surgical complications, length of hospital stay, and 30-day readmission to document the intervention’s financial impact and potential healthcare cost savings. Future studies should measure outcomes at the organizational and healthcare provider levels to provide a more comprehensive evaluation of the intervention implementability in the “real-world” clinical setting.

## Conclusion

To our knowledge, this is the first study proposing a group-based multimodal tele-prehabilitation intervention in a “real-world” clinical setting. Results of the current study suggest high acceptability, fidelity and feasibility, moderate benefits on physical function and physical activity behavior, and small decrease in stress level prior to cancer surgery.

## Data availability statement

The raw data supporting the conclusions of this article will be made available by the authors, without undue reservation.

## Ethics statement

The studies involving humans were approved by Comité d’éthique de la recherche du CHUM. The studies were conducted in accordance with the local legislation and institutional requirements. The participants provided their written informed consent to participate in this study.

## Author contributions

AP: Conceptualization, Formal Analysis, Investigation, Methodology, Project administration, Writing – original draft, Writing – review & editing, Data curation. DM: Validation, Writing – review & editing. SL: Validation, Writing – review & editing. ID: Validation, Writing – review & editing, Conceptualization, Funding acquisition, Supervision, Methodology.
